# Missing Teeth and Prosthetic Treatment in Patients Treated at College of Dentistry, University of Dammam

**DOI:** 10.1155/2017/7593540

**Published:** 2017-07-30

**Authors:** Shaimaa M. Fouda, Fahad A. Al-Harbi, Soban Q. Khan, Jorma I. Virtanen, Aune Raustia

**Affiliations:** ^1^Department of Substitutive Dental Sciences, College of Dentistry, University of Dammam, P.O. Box 1982, Dammam 31411, Saudi Arabia; ^2^Department of Clinical Affairs, College of Dentistry, University of Dammam, P.O. Box 1982, Dammam 31411, Saudi Arabia; ^3^Research Unit of Oral Health Sciences, Department of Community Dentistry, Faculty of Medicine, University of Oulu, P.O. Box 5281, 90014 Oulu, Finland; ^4^Medical Research Center Oulu, Oulu University Hospital and University of Oulu, Oulu, Finland; ^5^Research Unit of Oral Health Sciences, Department of Prosthetic Dentistry and Stomatognathic Physiology, Faculty of Medicine, University of Oulu, P.O. Box 5281, 90014 Oulu, Finland

## Abstract

The percentage of completely and partially edentulous patients and their prosthetic treatment at the Department of Substitutive Dental Sciences (SDS), College of Dentistry, University of Dammam, were investigated. Panoramic radiographs and medical records of adult patients (*n* = 479, mean age 45.9 years, and range 25–96 years) treated in 2011–2014 were examined. 6% of the patients were completely edentulous, 8% had single jaw edentulousness, and 74% were partially edentulous. Edentulousness was significantly correlated with age and the number of missing teeth was significantly higher among males (*p* < 0.026). Diabetes was significantly associated with complete edentulousness, single edentulous jaw (*p* value 0.015), and partial edentulousness (*p* value 0.023). Kennedy class III was the most frequent class of partial edentulousness in single and/or both jaws (*p* = 0.000). Patients having class I and/or class II were treated most often with removable partial dentures (RPD) (*p* = 0.000), while patients having class III were treated with fixed partial dentures (FPD). It was found that complete edentulousness increases in older age and the number of missing teeth was significantly higher among males. Kennedy class III was most common in both upper and lower jaw and was treated more often with FPD than with RPD.

## 1. Introduction

Tooth loss considerably reduces quality of life [1]. It causes impairment of speech, aesthetics, and mastication, as well as social impairment [1, 2]. It also has a negative impact on the patient's psychological status. It may cause depression and reduced self-esteem due to the loss of an important part of the body and an impaired self-image [1, 2].

Many factors lead to edentulousness, for example, caries and periodontal diseases, which are considered the main causes of tooth loss [3]. Other factors such as the quality of oral health services, socioeconomic status, educational level, smoking, area of residence, and pattern of dental visits are also related to edentulousness and play a significant role in its prevalence [1, 4, 5]. Edentulousness has been found to increase in old age, which may partly be attributable to physical disabilities that could occur in old age [1, 4]. Also, the prevalence of general and dental diseases increases with aging, leading to edentulousness [4].

Several studies have investigated the prevalence of edentulousness in relation to gender [4–8]. A higher percentage of edentulousness has been seen among females than among males [4, 6, 8]. Possible reasons for this are probably the higher prevalence of dental caries and periodontal diseases in women in addition to other social and economic reasons [8]. However, some studies have reported a higher incidence of edentulousness among males [5, 7, 9].

The prevalence of edentulousness has been used as an indicator to evaluate the efficiency of oral health services as well as to show the oral health of a population [10]. It has been monitored in several countries for decades [1, 4, 10]. The rate of edentulousness has declined particularly in the western countries, which is at least partly attributed to improved oral health services [11]. However, its prevalence is still high in some other countries [6, 11, 12]. Since the number of people advancing into old age is increasing worldwide, more edentulous people are expected, accordingly [2].

Although dental implants are increasingly used to support dental prosthesis, the conventional removable dentures are used in many cases due to financial and/or medical considerations. Patients demand to replace missing teeth is affected by several factors including availability of dental services and educational, financial, and social status of the patients [13, 14]. The demand for prosthodontic care is expected to increase among Saudis [14].

Limited data are available on the prevalence of edentulousness in the Arabic countries, as well as on predisposing factors. We investigated the percentage of completely and partially edentulous patients and their prosthetic treatment at the Department of Substitutive Dental Sciences (SDS), College of Dentistry, University of Dammam, in the Eastern Province of Saudi Arabia, and examined the relationship between tooth loss and age, gender, general health, and smoking. The hypothesis of the present study expected high level of edentulousness among the study group.

## 2. Materials and Methods

The proportion of completely and partially edentulous patients and their prosthetic treatment received at the Department of Substitutive Dental Sciences (SDS), College of Dentistry, University of Dammam, were investigated using panoramic radiographs and medical records. The total number of patients treated at the SDS Department between 2011 and 2014 was 1016, and of these the study group included 479 patients who were selected using the following inclusion criteria: age 25+ years, available panoramic radiograph, and complete patient record.

The number of male and female patients was 312 and 162, respectively. Their mean age was 45.9 years (SD 13.52, range 25–96 years). A majority of the patients who visited the SDS Department were Saudis (*n* = 295). According to the general authority of statistics of the Kingdom of Saudi Arabia, the number of residents in Dammam in 2010 was 914,493; 55,000 were Saudi residents and 365,000 were non-Saudis. The College of Dentistry, University of Dammam, is the only Dental College in the Eastern Province of the Kingdom of Saudi Arabia. It provides free charged treatment to the patients by the undergraduates (under supervision of faculty members), interns, and faculty staff members. The patients visiting the colleges' clinics are Saudi and from other nationalities who live in the Eastern Province, such as Philippines, Syrians, and Egyptians. The Department of SDS constitutes both subspecialties: removable and fixed prosthodontics.

### 2.1. Radiographic Evaluation

Panoramic radiographs of all the patients included in the study were examined by the same specialist dentist (S.F). Radiographs of the patients with all teeth missing in both jaws were recorded as edentulous; also the number of patients with a single edentulous jaw (maxillary or mandibular) was registered. Patients with at least one missing tooth other than the third molar were recorded as partially edentulous. Using the Kennedy classification, the number and location of missing teeth in the upper and lower jaw were categorized into four groups classes I, II, III, and IV [15].

The patients' medical records were reviewed and their age, gender, and nationality (Saudi/non-Saudi) were recorded. Data on the patients' medical condition were also recorded, including presence of diabetes and heart diseases. Patients' smoking habit was determined as smoker (currently smoking) or nonsmoker. The final prosthetic treatment that the partially edentulous patients received was determined to be a removable partial denture (RPD), a fixed partial denture (FPD), or both.

The proportion of completely edentulous patients, patients with a single edentulous jaw, and partially edentulous patients among the study group was determined. Also, a possible correlation between the number of missing teeth and the patient's age, gender, general health, and smoking habits was analyzed.

### 2.2. Statistics

Statistical Package of Social Sciences (SPSS version 19) was used for data entry and analysis. Cross tabulations and bar graphs were used to present descriptive statistics. For inferential statistics, Students'* t*-test, chi-square test, and binomial test were employed. Post hoc testing was employed to analyze the statistical significance between the prosthetic treatments provided to the patients with different Kennedy classes of partial edentulousness; significance was tested for each jaw separately.

## 3. Results

**Figure 1:**
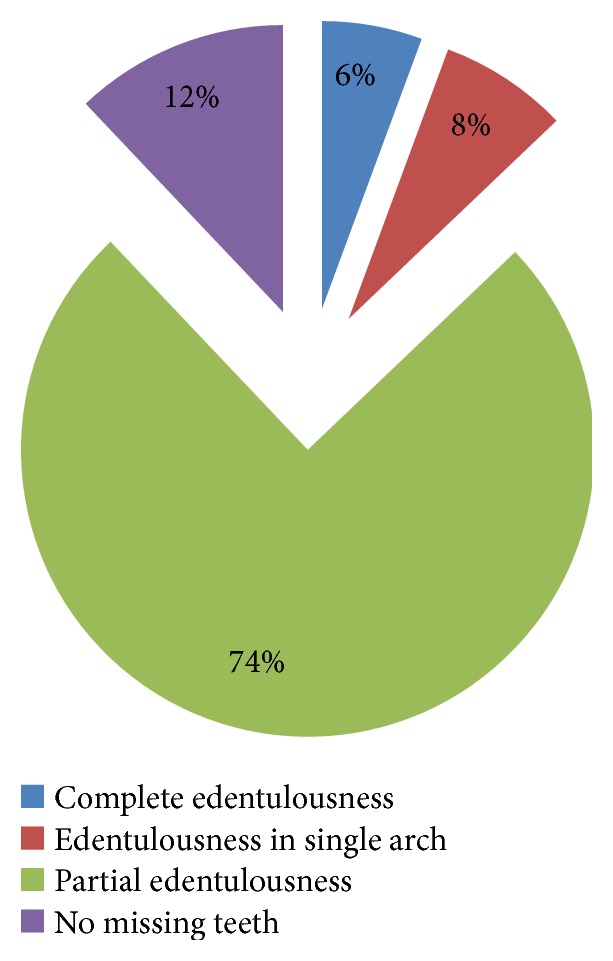
Percentage distribution of complete and partial edentulousness in patients (*n* = 479).

Most of the patients in the study group (*n* = 479) had missing teeth; 6% (*n* = 28) were completely edentulous, 8% (*n* = 37) had a single edentulous arch, 74% (*n* = 354) were partially edentulous, and 12% (*n* = 60) had no missing teeth (Figure 1).

**Figure 2:**
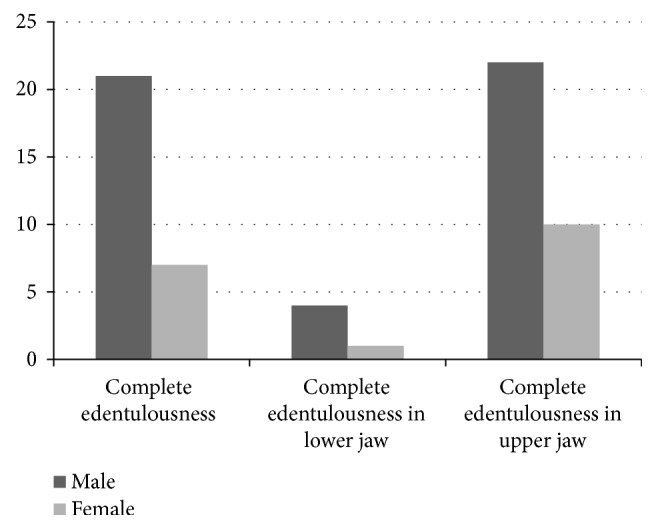
Frequency of complete edentulousness in relation to patients' gender.

Edentulousness correlated statistically significantly with age, being more common in older age (*p* value 0.000). All the completely edentulous patients and most of patients with a single edentulous jaw (34/37 patients) were 45 to 74 years old (Table 1). The mean age of completely edentulous patients was 63.7 (±11.3), while the remaining patients (*n* = 451) were 45.1 (±12.9) years old. No significant correlation was found between complete edentulousness and the patients' gender and nationality. Complete edentulousness in a single jaw was found to be significantly higher (*p* value 0.000) in the upper jaw (6%) than in the lower jaw (1%) and higher among males (70.3%) than among females (29.75%) (Figure 2). Edentulous maxilla and mandible were more common in males than in females (Figure 2).

The total number of completely edentulous patients and patients with edentulousness in a single jaw who had a general disease was 65, and almost one-third (*n* = 22) of them had diabetes, 4.6% had a heart disease, and 27.7% were smokers (Table 2). Only diabetes was found to be significantly related to complete edentulousness and edentulousness in a single jaw (*p* value 0.015).

In the partially edentulous group (*n* = 354), the average number of missing teeth was 6.5 (±5.3). Most of these patients (*n* = 197) missing 1–5 teeth were 25–34 years old (Table 1). The number of missing teeth was significantly higher among males (*p* < 0.026) than among females (Table 2). Partial edentulousness, Kennedy class III, was most usual in single and/or both jaws (*p* value 0.000) (Table 3).

Of the partially edentulous patients, 15% (*n* = 53) were diabetic, 4% (*n* = 14) had a heart disease, and less than one-quarter (*n* = 75) were smokers. On average, a diabetic patient had 8.1 (±6.7) missing teeth, compared with nondiabetic patients, who had 6.28 (±4.98); the difference in the mean was statistically significant (*p* value 0.023). Patients with a heart disease had an average of 6.79 (±5.89) missing teeth compared with patients with no heart disease 6.54 (±5.3); the difference in the mean was not statistically significant. Similarly, the mean number of missing teeth among smokers and nonsmokers was 7.07 (±5.26) and 6.39 (±5.34), respectively, with no significance in the mean difference (Table 2).

The number of patients who had received prosthetic treatment was 237. They had received fixed partial dentures (FPD), removable partial dentures (RPD), or both. One hundred and ten patients (31.1%) had received a RPD, one hundred and eleven (31.4%) a FPD, and sixteen patients (4.2%) both FPD and RPD. The patients with class I and/or class II in the upper and lower jaw were treated most often with a RPD (*p* value 0.000), while the number of FPD was significantly higher among patients with class III (Table 4).

## 4. Discussion

The prevalence of edentulousness has significantly decreased in North America and Europe [1, 4, 16]. However, a limited number of studies investigating the prevalence of edentulousness and related factors have been conducted in the Arabic region [9, 13, 17]. The prevalence of edentulousness among the studied group was 6%, which is much lower than in previous studies [4–7, 12]. A higher percentage of edentulousness of 13% and 17% has been reported among patients treated at a faculty hospital or dental clinics [7, 9].

The decreased percentage of edentulous patients among the study group might be because of the age distribution (25–96 years) among the studied sample, which included patients at younger age, compared with previous studies that have included only middle-aged and old patients. Also, it may result from the relatively small size of the sample in comparison with studies of national surveys. The results here are in line with some population based studies, although the sample size and selection are different [16, 18, 19]. A study from Mexico that included adults aged 18 years or more found that 6.3% were edentulous [18]. It was also found that 5% of UK adults between 55 and 64 years of age and 15% between 65 and 74 years are edentulous; these figures are in line with our findings [19]. Moreover, the prevalence of edentulousness among US adults aged 15 years or more reached 4.9% in 2009–2012 [16]. Conversely, a much lower prevalence of edentulousness has been reported in several African countries [17, 20, 21]. The prevalence of edentulousness in Ghana was reported to be 2.8% among people aged 50 years or more and 1.3% among adults aged 65 years or more in Ibadan Nigeria [20, 21].

Edentulousness was also found to be more frequent among older patients in our study. The highest percentage was observed in the age group above 70 (35.7%). It has been well established in the literature that edentulousness increases with age [4, 9]. The incidence of tooth loss has also been proven to be correlated with old age [22]. This is caused by many factors including difficulty to perform oral hygiene procedures due to systemic diseases or functional disability [23].

In the present study, a higher percentage of edentulousness was seen among males than among females. This finding is in line with the results of previous studies [5, 7, 13], one of them conducted in Saudi Arabia [9]. Regarding the partially edentulous patients, the number of missing teeth was significantly higher among males. A study conducted in Brazil found that men were more prone to lose teeth, which supports our result [22]. However, it disagrees with many studies that have found a higher rate of edentulousness among women [4, 8, 20]. In the present study, diabetes was found to be significantly correlated with partial and complete loss of teeth. Kennedy's class III was found to be the most prevalent class in the maxilla and mandible and also in both jaws together. This result agrees with several previous studies [24, 25].

To our knowledge, this study is the first in Dammam to investigate the percentage of partial and complete edentulousness and the prosthetic treatment of partially edentulous patients in a College of Dentistry, as well as the correlation between tooth loss and age, gender, general health, and smoking. However, the sample in the present study does not represent the population of the Eastern Province in Saudi Arabia, as it included patients seeking treatment at clinics of the College of Dentistry, University of Dammam only. Nevertheless, it provides baseline data for further studies. It also gives information about the most common prosthetic treatment for partially edentulous patients, which is beneficial for an educational institution. In addition, several previous studies have investigated the rate of edentulousness and associated factors among patients treated at faculty and outpatient clinics [7, 9, 13, 17]. The sample in the present study included 479 patients, which is close to the sample size of previous studies [7, 9]. The age of the patients ranged from 25 to 96 years, which is much lower than in most previous studies, which have investigated the prevalence of edentulousness among patients in middle and old age only [4–6]. However, several studies have investigated the rate of edentulousness among patients similar to the age group of the present study [9, 17, 18].

The strengths of this study include the fact that edentulousness was determined after screening the panoramic X-rays of all the participants. The X-rays were examined by the same dentist and did not depend on questionnaires filled in by the patients. However, a limitation is that the sample is not representative of the whole population but only includes patients treated at the College of Dentistry. In addition, the fact that panoramic radiographs were not available for all patients might have influenced the findings. A further study with a larger sample representing the whole population of the Eastern Province of Saudi Arabia will be needed.

## 5. Conclusions

Edentulousness was significantly more common in older age and the number of missing teeth was found to be higher among males than among females. Partial edentulousness, Kennedy class III, was most usual in single and/or both jaws. The patients with class I and/or class II in the upper and lower jaw were treated most often with RPD, while the number of FPD was significantly higher among patients with class III.

## Figures and Tables

**Table 1:** Number of missing teeth by age groups in partially edentulous patients (*n* = 354) treated in Dammam.

Missing teeth	Age (years)						Total
	25–34	35–44	45–54	55–64	65–74	75–84	
1–5	70	53	47	20	2	5	197
6–10	16	17	26	19	4	0	82
11–15	2	13	15	11	4	2	47
16–20	3	4	5	6	4	1	23
21–25	0	0	1	3	0	0	4
26–30	0	0	1	0	0	0	1
Total	91	87	95	59	14	8	354

**Table 2:** Number of missing teeth in partially edentulous patients (*n* = 354) treated in Dammam by gender, smoking, and general health.

	Number of missing teeth						Total
	1–5	6–10	11–15	16–20	21–25	26–30	
*Gender*							
Male	139 (61.5)	42 (18.6)	26 (11.5)	16 (7.1)	3 (1.3)	0	226
Female	58 (45.3)	40 (31.2)	21 (16.4)	7 (5.5)	1 (0.8)	1 (0.8)	128
*Smoking*							
Yes	38 (50.7)	20 (26.7)	11 (14.7)	6 (8)	0	0	75
No	159 (57.2)	61 (21.7)	37 (13)	17 (6.2)	4 (1.5)	1 (0.4)	279
*Diabetes*							
Yes	26 (49)	12 (22.6)	6 (11.3)	6 (11.3)	3 (5.7)	0	53
No	171 (56.7)	70 (23.3)	41 (13.7)	17 (5.7)	1 (0.3)	1 (0.3)	301
*Heart disease*							
Yes	7 (50)	4 (28.6)	1 (7.1)	2 (14.3)	0	0	14
No	189 (55.8)	79 (23)	46 (13.6)	21 (6.2)	4 (1.2)	1 (0.3)	340

Statistically significant with *p *value (<0.026).

**Table 3:** Distribution of Kennedy classes in maxillary and mandibular jaws in patients (*n* = 354) treated in Dammam.

Kennedy classes	Maxillary arch	Mandibular arch	Both arches
I	31 (15.2)	26 (12.5)	11 (7.7)
II	48 (23.5)	37 (17.8)	24 (16.8)
III	72 (35.3)	93 (44.7)	107 (74.8)
IV	7 (3.4)	3 (1.4)	1 (0.7)
NO missing teeth	46 (22.5)	49 (23.6)	0
*Total*	*204*	*208*	*143*

Post hoc test; statistically significant with *p *value = 0.000.

**Table 4:** Prosthetic treatment according to Kennedy classification in maxilla and mandible in patients (*n* = 237) treated in Dammam.

	RPD	FPD	RPD + FPD
*Kennedy classes Maxilla*			
I	24 (21.8)	5 (4.5)	0
II	27 (24.5)	12 (10.8)	3 (18.8)
III	51 (46.4)	71 (64)	11 (68.8)
IV	1 (0.9)	2 (1.8)	0
*Total*	*103*	*90*	*14*

*Mandible*			
I	22 (20)	4 (3.7)	3 (18.8)
II	32 (29.1)	18 (16.8)	5 (31.3)
III	44 (40)	63 (58.9)	8 (50)
IV	5 (4.5)	1 (0.9)	0
*Total*	*103*	*86*	*16*
